# Electrocardiographic findings in children and adolescents treated with antipsychotics: a cohort study

**DOI:** 10.3389/fpsyt.2026.1807504

**Published:** 2026-04-20

**Authors:** Fabio Pettinato, Alice Zanini, Adriana Prato, Federica Fiore, Nicoletta Maugeri, Rita Barone, Renata Rizzo

**Affiliations:** 1 Child and Adolescent Neurology and Psychiatric Section, Azienda Ospedaliera Universitaria Policlinico ‘G. Rodolico-San Marco’, Department of Clinical and Experimental Medicine, University of Catania, Catania, Italy; 2 Research Unit of Rare Diseases and Neurodevelopmental Disorders, Oasi Research Institute, IRCCS, Troina, Italy

**Keywords:** antipsychotics, cardiovascular safety, child psychiatry, electrocardiography, QTc interval prolongation, risk-based monitoring

## Abstract

**Background/objectives:**

Antipsychotic drugs are increasingly prescribed in children and adolescents across a wide range of psychiatric conditions. Although cardiovascular adverse effects are generally considered uncommon, concerns about electrocardiographic abnormalities, particularly QTc interval prolongation, have led to ongoing debate regarding appropriate monitoring strategies. Real-world data on the frequency, persistence, and clinical relevance of ECG findings during antipsychotic treatment in youth remain limited.

**Methods:**

This was a single-center, observational cohort study including patients younger than 18 years, treated with antipsychotics between January 2020 and December 2024. Inclusion required the availability of at least one 12-lead ECG performed during treatment and accompanied by a cardiology report. ECG parameters were extracted from all available recordings, with QTc calculated using Bazett’s formula and interpreted using sex-specific reference thresholds. ECG findings were analyzed primarily at the patient level, defining abnormalities based on their occurrence at any point during follow-up. An exploratory comparison was performed between patients with and without QTc prolongation.

**Results:**

The study included 430 patients (79.1% males; mean age 11.3 ± 3.35 years), of whom 429 had analyzable ECG data. At the patient level, 195 of 429 patients (45.5%) exhibited at least one numeric ECG abnormality during follow-up, most commonly heart rate abnormalities. QTc prolongation above sex-specific thresholds was observed in 24 patients (5.6%) and proved to be persistent in only 5 cases (20.8%), defined as occurrence in at least 2 ECG recordings. No patient exhibited a QTc ≥500 ms, and no clinically significant ventricular arrhythmias, high-grade conduction disturbances, or sudden cardiac events were observed. QTc prolongation was not significantly associated with sex, age, antipsychotic polypharmacy, combined first- and second-generation antipsychotic exposure, or QT-relevant comedications.

**Conclusions:**

In this large naturalistic pediatric cohort, ECG abnormalities during antipsychotic treatment were relatively frequent but predominantly mild, transient, and clinically benign. QTc prolongation occurred in a small minority of patients and was not associated with adverse cardiac outcomes. These findings may support a selective, risk-based approach to ECG monitoring in children and adolescents treated with antipsychotics, rather than routine universal screening.

## 1 Introduction

The use of antipsychotic (AP) drugs in pediatric populations has steadily increased over recent decades, with evidence of efficacy not only in schizophrenia spectrum disorders, but also in several non-psychotic conditions, including tic disorders, aggression, and psychomotor agitation. As a result, AP are frequently prescribed to children and adolescents for prolonged periods, often in the presence of multiple or complex neuropsychiatric diagnoses (1–4). Despite their clinical benefits, AP treatment in pediatric populations requires careful consideration due to a broad range of potential adverse effects, which include extrapyramidal symptoms, hyperprolactinemia, sedation, and metabolic alterations (5–7). Cardiovascular adverse effects have been less extensively studied, but remain clinically relevant, particularly in light of emerging evidence on long-term cardiometabolic risk (8–10). Population-based studies have investigated the incidence of cardiovascular adverse events among young individuals receiving psychotropic medications. In a nationwide register-based study from Sweden including more than 875,000 individuals aged 5–30 years exposed to psychopharmacological treatment, the mean annual incidence of cardiac events was approximately 0.99%, with arrhythmias accounting for about 0.51% of events and cardiac arrest remaining rare (around 0.01%). Incidence was higher among adolescent females and among patients exposed to psychotropic polypharmacy (11). Additional large pharmacoepidemiological studies further indicate that cardiovascular events associated with psychotropic medications in youth are generally uncommon but may vary according to demographic factors and treatment patterns, including age, sex, and combined medication exposure (12, 13). Within this context, increasing attention has been directed toward the cardiovascular safety of AP treatment in children and adolescents, especially with regard to electrocardiographic (ECG) changes and prolongation of the corrected QT interval (QTc) (8, 9, 14). ECG monitoring is a key component of safety assessment during AP treatment, as alterations in cardiac electrical activity may affect heart rate and ventricular repolarization (14, 15). In pediatric patients receiving AP, reported ECG abnormalities most commonly involve heart rate alterations, whereas QT interval prolongation appears to occur less frequently (16–18). QTc prolongation is considered a surrogate marker of increased susceptibility to malignant ventricular arrhythmias, although clinically significant events such as torsades de pointes remain rare (19–21). Interpretation of QTc values in children and adolescents is further complicated by the influence of heart rate and by variability in correction formulas. The Bazett formula remains commonly used in infants and young children, despite its tendency to overestimate QTc at higher heart rates; when this method is applied, a QTc value above 460 ms is generally considered clinically relevant (22). Although serious arrhythmias associated with QTc prolongation are uncommon, concerns about their potential severity have contributed to ongoing debate regarding the appropriate use of ECG monitoring in patients treated with AP. Available guidance and pediatric evidence consistently suggest that indiscriminate routine ECG monitoring is unlikely to be cost-effective, as QTc prolongation at presentation is relatively rare and more closely related to patient-specific factors than to antipsychotic exposure alone (23, 24). This underscores the importance of distinguishing clinically meaningful ECG abnormalities from incidental findings. Similar considerations regarding cardiovascular monitoring have also emerged in studies of other psychopharmacological treatments used in pediatric populations. A recent study evaluating guanfacine therapy in children and adolescents with ADHD reported modest changes in heart rate and QT interval without clinically meaningful QTc alterations (25). The present study describes a large real-world cohort of pediatric patients treated with AP who underwent repeated 12-lead ECG evaluations at a tertiary Child and Adolescent Neuropsychiatry service. Despite the growing body of literature on QTc changes during antipsychotic treatment in pediatric populations, large real-world cohorts with repeated ECG evaluations and longitudinal patient-level interpretation of ECG findings remain limited. Many available studies rely on relatively small samples, single ECG assessments, or controlled research settings that may not fully reflect routine clinical practice. The present study addresses this gap by analyzing ECG abnormalities and their persistence in a large naturalistic cohort of children and adolescents receiving antipsychotic treatment in routine clinical practice. The primary aim of this study was to characterize the frequency and nature of ECG findings during AP treatment, with particular attention to QTc prolongation and its interpretation in usual clinical care. A secondary aim was to exploratively examine associations between selected demographic or treatment-related factors and QTc prolongation. By providing a structured description of ECG findings in a naturalistic pediatric cohort, this study aims to contribute to a more balanced and evidence-based approach to cardiovascular monitoring in children and adolescents receiving AP treatment.

## 2 Materials and methods

### 2.1 Study design and data collection

This was an observational, single-center cohort study conducted at the Child and Adolescent Neuropsychiatry Section of the University Hospital of Catania (Italy). All patients younger than 18 years who were evaluated at our service and were receiving AP treatment for any indication between January 1, 2020, and December 31, 2024, were considered eligible. AP of any class and dosage were included. Patients were eligible if they received antipsychotic treatment during the study period and had at least one interpretable 12-lead ECG performed during treatment. Patients with known structural heart disease or congenital long QT syndrome were excluded, resulting in a study population primarily consisting of low-risk pediatric patients.

When available, ECGs obtained prior to the initiation of antipsychotic therapy were considered baseline examinations. ECGs were performed prospectively as part of routine clinical care either at baseline evaluation or during follow-up visits, with timing and frequency determined by clinical judgment and individual clinical indications (e.g. family history of cardiovascular disease, congenital or syndromic conditions, assumption of multiple medications). ECG recordings reflected routine cardiological monitoring and were not performed specifically for research purposes. Consequently, ECG monitoring was not systematically scheduled for all patients and the number of ECG evaluations could vary across individuals. For each patient, the observation period was defined as the interval between the first available ECG performed during antipsychotic treatment and the last ECG recorded within the study period. Follow-up duration therefore varied between patients depending on clinical monitoring practices and the availability of ECG recordings. Clinical, demographic, and pharmacological data were retrospectively extracted from medical records for the purposes of the present study. Information on neuropsychiatric diagnoses, syndromic or congenital conditions potentially associated with cardiovascular involvement, as well as family history of cardiovascular disease, was extracted from clinical records when available. Electrolyte abnormalities were defined according to local laboratory reference ranges and included hypomagnesemia, hypokalemia, and hypocalcemia when documented during the study period. These laboratory values were obtained as part of routine clinical monitoring during the antipsychotic treatment and were not systematically scheduled in relation to ECG recordings.

All patients and/or their legal guardians provided written informed consent for pharmacological treatment as part of standard clinical care. The consent procedure also included authorization for the use of anonymized clinical data for scientific research purposes in accordance with applicable regulations. The present study involved a retrospective analysis of fully anonymized clinical data collected during routine medical care. The study protocol was reviewed and approved by the Institutional Review Board of the University Hospital of Catania (prot. n. 67526, 03-12-2024). In accordance with institutional regulations, the requirement for additional informed consent for research purposes was waived due to the retrospective design of the study and the use of anonymized data.

### 2.2 Definition of pharmacological variables

Antipsychotic exposure was defined at the patient level as exposure to any first- or second-generation antipsychotic at any time during the observation period. Exposure categories were not mutually exclusive, as patients could receive more than one antipsychotic agent or switch between drug classes over time. Patients receiving more than one antipsychotic agent at any time during follow-up were classified as having antipsychotic polypharmacy. Exposure to both first- and second-generation antipsychotics during the observation period, whether sequential or concomitant, was defined as combined exposure. Given the observational design and the variability in ECG timing, exposure was not analyzed as a time-varying variable in relation to individual ECG recordings. Instead, exposure variables were summarized at the patient level and related to the occurrence of ECG findings during follow-up. Concomitant medications with potential relevance for QT interval modulation were defined as exposure during the study period to drug classes reported in the literature to influence cardiac repolarization or autonomic tone (26–28). These included selective serotonin reuptake inhibitors (SSRIs), serotonin-norepinephrine reuptake inhibitors (SNRIs), lithium, tetrabenazine, or methylphenidate. These medications were selected because they are commonly prescribed in pediatric neuropsychiatric populations and have been reported to influence cardiac electrophysiology either through direct effects on cardiac repolarization or through potential pharmacodynamic interactions with antipsychotic medications. Other medications with established QT-prolonging potential were not systematically prescribed in this cohort.

### 2.3 ECG acquisition and definition of ECG findings

12-lead ECGs were obtained either prior to the initiation of antipsychotic treatment (baseline evaluation) or during follow-up visits while patients were receiving AP treatment, according to clinical judgment and individual indications (e.g., initiation or modification of pharmacological treatment, presence of comorbid conditions, or clinical monitoring). ECGs were not systematically scheduled at predefined time points and were not performed specifically for research purposes.

The observation period was defined at the patient level as the interval between the first available ECG recorded during antipsychotic treatment and the last ECG performed within the study period (January 1, 2020 to December 31, 2024). When available, ECGs obtained prior to antipsychotic initiation were considered baseline examinations. Follow-up duration and number of ECG recordings therefore varied across patients depending on clinical monitoring practices.

ECGs were recorded using a GE Healthcare MAC 2000 electrocardiograph and interpreted by pediatric cardiologists as part of routine clinical care. ECG tracings were not re-evaluated specifically for the purposes of this study. Patients without interpretable ECG tracings were excluded from ECG-level analyses.

From each ECG, the following parameters were extracted: heart rate, RR interval, PR interval, QRS duration, and corrected QT (QTc) interval. QTc was automatically calculated using Bazett’s formula, in accordance with routine clinical practice and the majority of pediatric ECG reference studies. Although alternative correction formulas (e.g., Fridericia or Framingham) have been proposed, Bazett’s correction was used to ensure consistency across ECG recordings and comparability with established pediatric reference values.

ECG abnormalities were defined according to established pediatric electrocardiographic reference standards derived from large population-based studies (29, 30). QTc prolongation was defined using sex-specific thresholds (≥460 ms in males and ≥470 ms in females), reflecting commonly adopted clinical reference limits that extend percentile-based normative data. The primary analysis of QTc prolongation was based on sex-specific thresholds. For descriptive comparison, an additional analysis using a fixed threshold of ≥460 ms irrespective of sex was also performed. Persistent QTc prolongation was defined as the occurrence of QTc prolongation in at least two separate ECG recordings during follow-up. Heart rate abnormalities (tachycardia and bradycardia) were defined according to age-adjusted pediatric reference ranges. Conduction abnormalities were defined using pragmatic thresholds consistent with upper limits reported in pediatric populations, including QRS prolongation (>100 ms) and PR interval prolongation (>180 ms), particularly applicable to school-aged children and adolescents. PR interval shortening (<120 ms) was explored descriptively but was not classified as an ECG abnormality, as values below this threshold are commonly observed as physiological variants in pediatric populations.

Qualitative ECG findings reported by the cardiologist (e.g., incomplete right bundle branch block or nonspecific intraventricular conduction delay) were recorded and analyzed descriptively, as they were not predefined quantitative outcomes.

Analyses were primarily conducted at the patient level, defining the presence of an ECG finding as the occurrence of at least one abnormal ECG parameter during the observation period. ECG-level data were used for descriptive purposes only, and ECG findings were not modeled in relation to the timing or dose of antipsychotic exposure. Given the observational design, the study was not intended to assess causal or time-dependent relationships between antipsychotic exposure and ECG changes.

### 2.4 Statistical analysis

Statistical analyses were performed using IBM SPSS Statistics version 29.0 (IBM Corp., Armonk, NY, USA). Continuous variables were summarized as mean ± standard deviation (SD) or median with interquartile range (IQR), as appropriate. Categorical variables were expressed as counts and percentages. The prevalence of ECG findings was calculated at the patient level. For each participant, ECG abnormalities were defined as the occurrence of at least one abnormal value in any ECG recording during the observation period. QTc interval characteristics were further described using the maximum QTc value observed for each patient during follow-up. For QTc-related analyses, patients were classified according to the occurrence of QTc prolongation at any time during follow-up. Specifically, QTc prolongation was defined as the presence of at least one ECG recording exceeding the predefined sex-specific threshold, regardless of whether the finding was transient or persistent across subsequent ECGs. Given the observational design and the variability in ECG timing, ECG findings were not analyzed as time-dependent variables, and transitions between normal and prolonged QTc values over time were not modeled. An exploratory comparative analysis was performed between patients with and without QTc prolongation to evaluate potential clinical and treatment-related associations, including sex, age, antipsychotic polypharmacy, exposure to first- and second-generation AP, and use of concomitant medications with potential relevance for QT interval modulation. Comparisons of categorical variables were conducted using Pearson’s chi-square test or Fisher’s exact test, as appropriate. A regression-based approach was not applied due to the limited number of events and the exploratory nature of the analysis, which was not designed to identify independent predictors but rather to describe potential associations. All statistical tests were two-tailed, and a p-value < 0.05 was considered statistically significant. Missing data for the primary ECG parameters were minimal and were not imputed.

## 3 Results

### 3.1 Sample characteristics

Demographic and clinical characteristics of the sample are summarized in **Table 1**. The study population comprised 430 pediatric patients (340 males, 79.1%). At the beginning of data collection, the mean age of the cohort was 11.3 ± 3.35 years (range 4–18 years). The majority of patients presented with multiple neuropsychiatric conditions, with 350 patients, 81.4%, presenting with two or more neuropsychiatric diagnoses. The most frequently documented diagnoses were conduct disorder (282 patients, 65.6%), intellectual disability (185 patients, 43.0%), and tic disorders or Tourette syndrome (172 patients, 40.0%). Syndromic or congenital conditions potentially associated with cardiovascular involvement were identified in 20 patients (4.7%). A family history of cardiovascular disease was reported in 107 patients (24.9%). Electrolyte abnormalities identified in routine laboratory tests during the observation period were uncommon: hypomagnesemia was observed in 5 patients (1.2%), while no cases of hypocalcemia or hypokalemia were documented in the laboratory tests available.

**Table 1:** Demographic and clinical characteristics of the study population (n = 430).

Demographics	
Age, years, mean ± SD (range)	11.28 ± 3.35 (4 – 18)
Male sex, n (%)	340 (79.1)
Neuropsychiatric diagnoses, n (%)	
Conduct disorder	282 (66)
Intellectual disability	185 (43)
Tic disorders/Tourette syndrome	172 (40)
Obsessive–compulsive disorder	107 (25)
Autism spectrum disorder	92 (21)
Attention-deficit/hyperactivity disorder	70 (16)
Anxiety disorders	30 (7)
Psychotic disorders	24 (6)
Psychiatric comorbidity	
≥2 diagnoses, n (%)	350 (81)
Medical and family history	
Syndromic or congenital condition, n (%)	20 (5)
Family history of cardiovascular disease, n (%)	107 (25)
Electrolyte abnormalities during the observation period	
Hypomagnesemia, n (%)	5 (1)
Hypocalcemia, n (%)	0 (0)
Hypokalemia, n (%)	0 (0)

Age refers to the patient’s age at the first available ECG during the study period. Percentages may exceed 100% because patients could have multiple diagnoses.

### 3.2 Antipsychotic treatment characteristics

The majority of patients were treated with second-generation AP. Overall, 414 patients (96.3%) received at least one second-generation AP during the observation period, while 64 patients (14.9%) were exposed to first-generation AP. Combined exposure to both second- and first-generation AP was observed in 48 patients (11.2%). Because patients could receive more than one antipsychotic medication during the observation period, exposure categories were not mutually exclusive, and some patients contributed to both first- and second-generation antipsychotic exposure groups. AP polypharmacy, defined as treatment with two or more antipsychotic agents, was documented in 100 patients (23.3%). Risperidone was the most frequently prescribed AP (334 patients, 77.7%), followed by aripiprazole (114 patients, 26.5%). First-generation AP were prescribed less frequently, with promazine (25 patients, 5.8%) and haloperidol (17 patients, 4.0%) being the most commonly used among this class. Among the most frequently prescribed AP, the median daily dose during the observation period was 1.0 mg (interquartile range [IQR] 1.0) for risperidone and 5.0 mg (IQR 7.5) for aripiprazole. At baseline, 97 patients (22.6%) were receiving at least one concomitant medication with potential relevance for QT interval modulation.

### 3.3 Prevalence of ECG findings

One patient was excluded from ECG-level analyses due to the absence of interpretable ECG data. Consequently, 2040 ECG recordings from 429 patients were included in the analysis (**Table 2**). Baseline ECG recordings prior to antipsychotic initiation were available in 383 patients (89.3%) and were used for descriptive purposes only. During follow-up, patients underwent a median of 4 ECG examinations (IQR 3–6), with a median ECG follow-up duration of 1.5 years (IQR 0.72–2.79). At the patient level, 195 out of 429 patients (45.5%; 95% CI 40.8–50.2) exhibited at least one numeric ECG finding during the observation period. The most frequently observed findings were heart rate abnormalities, with tachycardia documented in 90 patients (21.0%) and bradycardia in 70 patients (16.3%). A prolonged QTc interval above sex-specific reference thresholds was identified in 24 patients (5.6%; 95% CI 3.8–8.2). QRS complex prolongation (>100 ms) was observed in 38 individuals (8.9%), while prolonged atrioventricular conduction (PR interval >180 ms) was less common, occurring in 9 patients (2.1%).

**Table 2:** Prevalence of ECG findings at the patient level (n = 429).

ECG parameter	n (%) or median (IQR)
Number of ECG recordings per patient, median (IQR)	4 (3–6)
Any numeric ECG finding	195 (45.5; 95% CI 40.8–50.2)
QTc prolongation above sex-specific thresholds	24 (5.6; 95% CI 3.8–8.2)
Tachycardia	90 (21.0)
Bradycardia	70 (16.3)
QRS prolongation (>100 ms)	38 (8.9)
PR prolongation (>180 ms)	9 (2.1)

A total of 2040 ECG recordings were analyzed across the study population. Numeric ECG findings refer to predefined quantitative abnormalities in heart rate, PR interval, QRS duration, or QTc interval. QTc prolongation was defined using sex-specific reference thresholds (≥460 ms in males and ≥470 ms in females). QRS prolongation was defined as >100 ms and PR prolongation as >180 ms. Tachycardia and bradycardia were defined according to age-adjusted pediatric reference ranges.

### 3.4 QTc interval findings

Among the 429 patients with available ECG data, the maximum QTc value per patient showed a median of 435 ms (IQR 26; range 371–489). When using a fixed threshold of ≥460 ms irrespective of sex, QTc prolongation was observed in 33 patients (7.7%). Among these patients, QTc prolongation was persistent in 5 cases (20.8%), defined as the occurrence of QTc prolongation in two or more ECG recordings. In the remaining patients, QTc prolongation was classified as transient, as it was observed in only one ECG recording and not confirmed in subsequent tracings. QTc prolongation occurred as an isolated finding, in the absence of concomitant heart rate or conduction abnormalities in 12 patients (50.0%), while the other cases were associated with additional ECG findings.

### 3.5 Exploratory analysis of QTc prolongation

An exploratory comparison was performed between patients who exhibited QTc prolongation during follow-up and those who did not (**Table 3**). No significant differences were observed with respect to sex, AP polypharmacy, combined exposure to first- and second-generation AP, or the use of concomitant medications with potential relevance for QT interval modulation (all p values > 0.05). Patients with QTc prolongation showed a similar age distribution compared with those without QTc prolongation (mean age 10.9 ± 2.5 vs 11.3 ± 3.4 years; median age 11 years in both groups). Overall, this exploratory analysis did not identify clear clinical or treatment-related factors associated with QTc prolongation in the study cohort.

**Table 3:** Exploratory comparison of patients with and without prolonged QTc (defined according to sex-specific thresholds) (n = 429).

Variable	Prolonged QTc (n = 24)	No prolonged QTc (n = 405)	p-value
Age, years, mean ± SD	10.9 ± 2.5	11.3 ± 3.4	–
Female sex, n (%)	7 (29.2)	83 (20.5)	0.307
Antipsychotic polypharmacy, n (%)	4 (16.7)	96 (23.7)	0.619
Combined SGA and FGA exposure, n (%)	2 (8.3)	46 (11.4)	1.000
QT-relevant comedication, n (%)	6 (25.0)	91 (22.5)	0.802

Comparative analyses were exploratory; p-values were not adjusted for multiple testing. Age was summarized descriptively due to the limited number of patients with prolonged QTc.

### 3.6 Persistence and clinical relevance of other ECG findings

The majority of ECG findings other than QTc prolongation were mild, transient, and clinically nonspecific. Qualitative ECG findings reported by the cardiologist, such as incomplete right bundle branch block or nonspecific intraventricular conduction delay, were also observed; however, these findings were generally interpreted as incidental or constitutional and did not show progressive changes during follow-up. Heart rate abnormalities, including tachycardia and bradycardia, represented the most frequently observed findings and were often intermittent across follow-up evaluations, as they were not consistently present in repeated ECG recordings for the same patient. Conduction abnormalities, such as QRS or PR interval prolongation, were less common and generally did not persist across multiple ECG recordings. No patient developed high-grade atrioventricular block, sustained ventricular arrhythmias, or other clinically significant rhythm disturbances during the observation period. Importantly, no ECG finding required urgent discontinuation of AP treatment, emergency cardiological intervention, or hospitalization. When cardiological evaluation was requested, ECG alterations were interpreted as benign or non-clinically relevant, and continuation of the prescribed pharmacological regimen was recommended in all cases.

## 4 Discussion

In this large naturalistic cohort of children and adolescents treated with AP medications, ECG abnormalities were frequently observed, but were predominantly mild, transient, and clinically benign in nature. Nearly half of the patients exhibited at least one numerical ECG abnormality during follow-up, most commonly involving heart rate parameters. Heart rate abnormalities, which represented the most frequent ECG findings in our cohort, should be interpreted in light of normal pediatric physiological variability. In children and adolescents, heart rate is highly influenced by autonomic tone, emotional state, and situational factors, and transient deviations from age-adjusted reference ranges are common and often not clinically meaningful (29–31). In this context, the predominance of heart rate changes observed in our study likely reflects physiological variability rather than a direct pharmacological effect of antipsychotic treatment. QTc interval prolongation beyond sex-specific reference thresholds occurred in a small proportion of patients (5.6%), was usually transient, and persisted in only a minority of cases. No clinically significant ventricular arrhythmias, high-grade conduction disturbances, sudden cardiac events, or AP-related hospitalizations occurred. Importantly, ECG findings did not prompt urgent discontinuation of antipsychotic treatment, and when cardiological consultation was requested, continuation of therapy was consistently recommended.

Overall, the frequency and characteristics of ECG findings in our cohort are consistent with previous pediatric literature indicating a low prevalence of clinically relevant QTc prolongation during AP treatment. Prospective and observational studies in children and adolescents generally report stable QTc values over time and a very low proportion of values exceeding thresholds associated with increased arrhythmic risk (16–18, 32). Naturalistic longitudinal data further suggest that modest QTc changes are more strongly associated with individual vulnerability factors, such as baseline cardiometabolic status, than with AP exposure itself (23, 33). Our findings extend this evidence by providing a large real-world pediatric cohort with repeated longitudinal ECG assessments, allowing a detailed patient-level characterization of ECG abnormalities during antipsychotic treatment. These observations are also consistent with a recent systematic review by Gasparini et al. (9), which reported that commonly prescribed second-generation antipsychotics in pediatric populations, particularly risperidone and aripiprazole, are generally associated with minimal or no clinically significant QTc prolongation. The review also highlighted the limited number of available pediatric studies and the lack of standardized ECG monitoring protocols, emphasizing the need for further research in this area.

QTc prolongation is commonly considered a surrogate marker of arrhythmic risk; however, its clinical interpretation in pediatric populations requires caution. Physiological variability related to heart rate, autonomic tone, and circadian factors may influence QTc values in children and adolescents. QTc intervals in adolescents receiving psychotropic medications may also become more pronounced under dynamic conditions, such as after rising rapidly from the supine position, highlighting the potential value of complementary ECG assessments beyond resting measurements (34). Consequently, the clinical interpretation of isolated QTc findings should be cautious, and repeated ECG measurements or complementary assessments (such as ambulatory ECG monitoring) may provide additional clinical context. In clinical practice, arrhythmic risk is generally considered more relevant when QTc values exceed higher thresholds, typically ≥500 ms (19). The QTc values observed in our cohort, while occasionally exceeding reference limits, remained well below thresholds associated with increased arrhythmic risk and were not accompanied by adverse clinical outcomes. However, it should be acknowledged that rare but serious cardiac events may not be fully captured in observational cohort studies. Continued pharmacovigilance and long-term cardiovascular monitoring therefore remain important components of the overall safety assessment of antipsychotic treatment in pediatric populations. Current evidence and guidelines support a selective, risk-based approach to ECG monitoring, based on individual clinical risk factors (15, 20, 24, 35). Our data are consistent with this risk-based framework. Despite repeated ECG evaluations in a large and clinically complex cohort, no actionable findings emerged that required urgent intervention. Real-world studies examining monitoring practices in pediatric outpatient settings similarly report substantial heterogeneity and low rates of ECG monitoring, without evidence that systematic screening improves clinical outcomes (6).

The strengths of this study include the large sample size, the naturalistic real-world design, and the availability of repeated ECG recordings interpreted by pediatric cardiologists as part of routine care. Patient-level longitudinal analysis allowed for a clinically meaningful assessment of the persistence and context of ECG abnormalities, rather than reliance on isolated measurements. Additionally, the cohort reflects contemporary prescribing practices, with predominant use of second-generation AP at low to moderate doses, as reflected by the median daily doses of risperidone and aripiprazole (1.0 mg and 5.0 respectively). Nonetheless, several limitations should be acknowledged. The study was conducted in a single tertiary care center and included a high proportion of patients with multiple neuropsychiatric comorbidities. These characteristics may limit the generalizability of the findings to other clinical settings, particularly community-based populations. However, they also reflect the clinical complexity typically encountered in specialized child and adolescent neuropsychiatry services, where antipsychotics are most frequently prescribed. The observational design precludes causal inference. ECG timing and frequency were determined by routine clinical practice rather than by a standardized research protocol, which may have introduced variability in the timing of ECG recordings across patients. Because ECG monitoring was performed according to routine clinical practice rather than a predefined study protocol, the possibility of selection bias cannot be excluded. In particular, patients perceived as having a higher cardiovascular risk may have undergone more frequent ECG evaluations, potentially influencing the observed frequency of ECG findings. QTc values were derived using Bazett’s formula. Bazett’s formula is known to overestimate QTc values particularly at higher heart rates, which are common in pediatric populations. As a result, some modest QTc prolongations observed in our cohort may partly reflect methodological overcorrection rather than true repolarization abnormalities. Alternative correction formulas, such as Fridericia’s formula, have been proposed to reduce heart rate-related bias; however, Bazett’s formula remains the most commonly used correction method in pediatric cardiology and ensured consistency across ECG recordings in our clinical setting. In addition, the study population largely consisted of patients without known structural heart disease, which may partly explain the limited occurrence of clinically significant ECG findings. Finally, although the absence of serious arrhythmic events limits conclusions regarding rare outcomes, this finding is consistent with the known epidemiology of AP-related arrhythmias in pediatric populations. Detailed information on AP and comedications dose, duration of treatment, and cumulative exposure was not consistently available across patients due to the retrospective and naturalistic design of the study. AP and comedications often varied during follow-up according to clinical needs, which limited the possibility of performing reliable exposure-based or dose–response analyses. Future prospective studies with standardized dose and longitudinal exposure assessment would be better suited to investigate potential dose-response relationships between AP treatment, comedications and ECG findings.

In conclusion, ECG abnormalities during AP treatment in children and adolescents in our cohort were numerically frequent but rarely clinically significant. QTc prolongation below high-risk thresholds was not associated with adverse outcomes in this large cohort of predominantly low-risk pediatric patients. These findings support consideration of a balanced, risk-based approach to ECG monitoring, prioritizing careful clinical assessment and targeted surveillance over universal screening, with the aim of optimizing cardiovascular safety while preserving access to effective AP treatment.

## Data Availability

The raw data supporting the conclusions of this article will be made available by the authors, without undue reservation.
